# Effectiveness of a Therapeutic *Tai Ji Quan* Intervention vs a Multimodal Exercise Intervention to Prevent Falls Among Older Adults at High Risk of Falling: A Randomized Clinical Trial

**DOI:** 10.1001/jamainternmed.2018.3915

**Published:** 2018-09-10

**Authors:** Fuzhong Li, Peter Harmer, Kathleen Fitzgerald, Elizabeth Eckstrom, Laura Akers, Li-Shan Chou, Dawna Pidgeon, Jan Voit, Kerri Winters-Stone

**Affiliations:** 1 Oregon Research Institute, Eugene; 2 School of Kinesiology, Shanghai University of Sport, Shanghai, China; 3 Department of Exercise and Health Science, Willamette University, Salem, Oregon; 4 Oregon Medical Group, Eugene, Oregon; 5 Division of General Internal Medicine and Geriatrics, Oregon Health & Science University, Portland; 6 Department of Human Physiology, University of Oregon, Eugene; 7 Department of Rehabilitation, Dartmouth-Hitchcock Medical Center, Lebanon, New Hampshire; 8 Voit Better Balance, Mercer Island, Washington; 9 Knight Cancer Institute and School of Nursing, Oregon Health & Science University, Portland

## Abstract

**Importance:**

Falls in older adults are a serious public health problem associated with irreversible health consequences and responsible for a substantial economic burden on health care systems. However, identifying optimal choices from among evidence-based fall prevention interventions is challenging as few comparative data for effectiveness are available.

**Objective:**

To determine the effectiveness of a therapeutically tailored *tai ji quan* intervention, *Tai Ji Quan*: Moving for Better Balance (TJQMBB), developed on the classic concept of *tai ji* (also known as tai chi), and a multimodal exercise (MME) program relative to stretching exercise in reducing falls among older adults at high risk of falling.

**Design, Setting, and Participants:**

A single-blind, 3-arm, parallel design, randomized clinical trial (February 20, 2015, to January 30, 2018), in 7 urban and suburban cities in Oregon. From 1147 community-dwelling adults 70 years or older screened for eligibility, 670 who had fallen in the preceding year or had impaired mobility consented and were enrolled. All analyses used intention-to-treat assignment.

**Interventions:**

One of 3 exercise interventions: two 60-minute classes weekly for 24 weeks of TJQMBB, entailing modified forms and therapeutic movement exercises; MME, integrating balance, aerobics, strength, and flexibility activities; or stretching exercises.

**Main Outcomes and Measures:**

The primary measure at 6 months was incidence of falls.

**Results:**

Among 670 participants randomized, mean (SD) age was 77.7 (5.6) years, 436 (65%) were women, 617 (92.1%) were white, 31 (4.6%) were African American. During the trial, there were 152 falls (85 individuals) in the TJQMBB group, 218 (112 individuals) in the MME group, and 363 (127 individuals) in the stretching exercise group. At 6 months, the incidence rate ratio (IRR) was significantly lower in the TJQMBB (IRR, 0.42; 95% CI, 0.31-0.56; *P* < .001) and MME groups (IRR, 0.60; 95% CI, 0.45-0.80; *P* = .001) compared with the stretching group. Falls were reduced by 31% for the TJQMBB group compared with the MME group (IRR, 0.69; 95% CI, 0.52-0.94; *P* = .01).

**Conclusions and Relevance:**

Among community-dwelling older adults at high risk for falls, a therapeutically tailored *tai ji quan* balance training intervention was more effective than conventional exercise approaches for reducing the incidence of falls.

**Trial Registration:**

ClinicalTrials.gov identifier: NCT02287740

## Key Points

**Question:**

Is a fall prevention–specific *tai ji quan* intervention clinically more effective in reducing falls among older adults at high risk of falling than a stretching intervention (control) or a standard multimodal exercise intervention?

**Findings:**

In a randomized clinical trial involving 670 adults 70 years or older with a history of falls or impaired mobility, the therapeutic *tai ji quan* intervention effectively reduced falls by 58% compared with the stretching exercise (control intervention) and by 31% compared with a multimodal exercise intervention.

**Meaning:**

For older adults at high risk of falling, a therapeutically tailored *tai ji quan* intervention was more effective than stretching or multimodal exercises in reducing the incidence of falls.

## Teaser

This randomized clinical trial compares the effectiveness of a *tai ji quan* intervention with multimodal exercise and stretching exercise in reducing falls in older adults who had fallen in the past year.

## Introduction

Falls in older adults constitute a major public health problem in the United States.^1,2^ Annually, approximately 28% of community-dwelling adults 65 years or older report falling; an estimated 38% of these falls result in injuries^2^ leading to emergency department visits, hospital admissions, or death.^3,4^ Fall-related treatments are costly, averaging $9389 per fall for fall-related injuries among Medicare beneficiaries.^5^ In 2015, the total medical costs for falls in persons aged 65 years and older were more than $50 billion, 75% of which fell to Medicare/Medicaid.^6^

Falls, however, are largely preventable, with mounting evidence suggesting that exercise can be a safe and effective way to reduce falls.^7,8^ However, identifying optimal choices from among available evidence-based fall prevention interventions is challenging because few comparative effectiveness data are available, especially for older adults with high fall risk.^9^ With the continuing growth of the older segment of the population^10^ and the concomitant projected increase in the number of falls,^2^ high health care spending,^11^ and escalating health care costs,^12^ identifying the exercise intervention that is the most safe, effective, and easily implementable would greatly aid clinicians and health care institutions in making informed decisions about which interventions to prescribe given clinical goals and fiscal constraints.

This trial was designed to respond to this evidence gap and these clinical decision needs. We aimed to determine the comparative effectiveness of 2 proven interventions, therapeutically tailored *tai ji quan* exercise (*Tai Ji Quan*: Moving for Better Balance [TJQMBB])^13,14,15^ and multimodal exercise,^16^ relative to stretching exercise in reducing the incidence of falls in older adults at high risk of falling. Our primary hypothesis for this trial was that, compared with stretching or multimodal exercise programs, TJQMBB would be clinically more effective in reducing the number of falls.

## Methods

### Study Design

We performed a single-blind, parallel-design, randomized clinical trial with participants randomly allocated to 1 of 3 active arms: TJQMBB, entailing modified *tai ji quan* forms (derived from the classic framework of *tai ji*, also known as tai chi) and associated therapeutic movement exercises; multimodal exercise, integrating aerobic, strength, balance, and flexibility activities; or stretching exercises (the control arm) (Figure). The trial protocol (available in the Supplement) was approved by the institutional review board of Oregon Research Institute, and an independent data and safety monitoring board appointed by the National Institute on Aging oversaw the study. Written informed consent was obtained from all participants.

**Figure.**
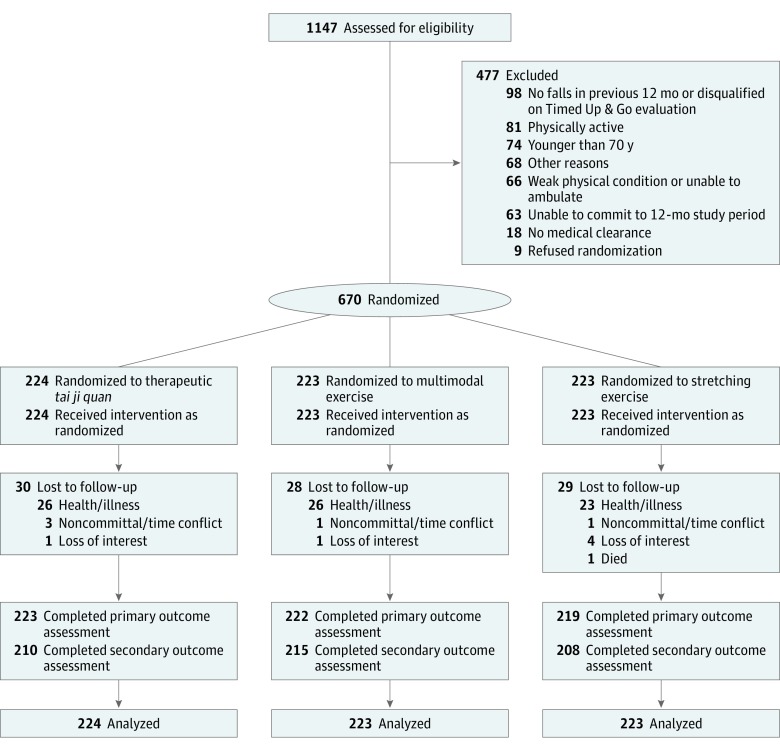
Flow of Participants Through the Trial

### Population, Setting, and Recruitment

The target population was community-dwelling older adults living in 7 urban and suburban cities across 3 counties in Oregon. These counties were strategically chosen because of a moderate to high density of older adult populations and a high incidence of fall injuries.^17^ Eligible participants were 70 years or older and met one of the following primary criteria: (1) having fallen at least once in the preceding 12 months and having a health care practitioner’s referral indicating that the participant was at risk of falls or (2) having impaired mobility as evidenced by a Timed Up & Go (TUG)^18^ result greater than 13.5 seconds.^19^ Other inclusion criteria were as follows: (1) ability to walk 1 or 2 blocks, with or without the use of an assistive device; (2) ability to exercise safely as determined by a health care practitioner; and (3) willingness to be randomly assigned to and complete a 6-month intervention. We excluded individuals who had (1) participated in daily or structured vigorous physical activity or walking for exercise that lasted 15 minutes or longer or muscle-strengthening activities on 2 or more days a week in the previous 3 months, (2) severe cognitive impairment (Mini-Mental State Examination^20^ score, ≤20 on a range of 0 to 30), or (3) major medical or physical conditions determined by their health care practitioner to preclude exercise.

Recruitment strategies included promotions at local senior or community centers, senior meal sites, medical clinics, statewide senior falls prevention networks, targeted mass mailings, and local newspaper advertisements. Recruitment lasted from February 20, 2015, to August 29, 2018, with the final participant follow-up on January 30, 2018.

### Randomization and Masking

Eligible older adults were randomly assigned in a 1:1:1 ratio to receive 1 of the 3 interventions via a computer-generated randomization sequence with a block size of 3 or 6 to prevent anticipation of assignment to study condition. Because this was a behavioral intervention, study participants were not blinded to intervention group allocation. Primary and secondary outcome assessors were masked to group allocation and remained separate from the intervention team, and class instructors (interventionists) were blinded to the study’s hypothesis.

### Interventions and Procedures

Each of the 3 interventions involved a 60-minute exercise session twice weekly for 24 weeks. In all 3 groups, each session consisted of a 10-minute warm-up, 40 to 45 minutes of core exercises, and a 5-minute cool-down activity. Exercise intensity in each intervention group was monitored through a subjective measure of perceived exertion (Exercise Intensity section of Trial Protocol in the Supplement). Intervention classes varied in size, with a range of 9 to 21 participants, and were held in community facilities, such as senior or community centers, churches, or nonprofit organizations. All 3 interventions were conducted concurrently and delivered in 15 class sites throughout the study area. At each site, the 3 interventions were separated in time to avoid cross-contamination.

#### Therapeutic *Tai Ji Quan*

The training protocol, *Tai Ji Quan*: Moving for Better Balance (see the Supplement for detail), involved practice of a core of 8 therapeutically modified exercise forms with built-in variations and a subroutine of integrated therapeutic movement exercises.^14,15,21^ Aimed at stimulating and integrating musculoskeletal, sensory, and cognitive systems, the practice focused on controlled, self-initiated *tai ji quan*–based exercises with synchronized breathing, including center of gravity displacement using a dynamic interplay of stabilizing and self-induced destabilizing postural actions involving unilateral weight-bearing and weight-shifting movements, trunk and pelvic rotation, ankle sway, and eye-head-hand movements.^21^

During the initial 10 weeks, sessions focused on learning and performing the TJQMBB forms in various formats (ie, seated, standing in place, and stepping), accompanied by sets of therapeutic and functional tai ji quan–based exercises involving ankle sway, sit-to-stand, single-leg stands, turning, and stepping exercises(referred to as mini-therapeutic movements).^21^ At each session, participants practiced 3 to 4 sets of a *tai ji quan* form, with 3 to 5 repetitions in each set intermingled with 3 to 5 sets of 3 to 4 selected mini-therapeutic movements (4 to 5 repetitions in each set). After all 8 therapeutic *tai ji quan* forms had been learned (weeks 11 and 12), each session comprised 5 to 6 sets of variations in the 8-form routine and 3 to 4 mini-therapeutic movements in sets of 4 to 5.

#### Multimodal Exercise

The training protocol involved aerobic conditioning, strength, balance, and flexibility activities.^16^ The aerobic exercises included long strides, heel-toe walking, narrow- and wide-based walking, and sidestepping for cardiovascular fitness. Strength training included exercises for ankle dorsiflexors, knee extensors, and hip abductors. Balance training involved tandem foot-standing, heel-toe and line walking, single-leg standing, alternation of the base of support, weight transfers, and various reaching movements away from the center of gravity. Flexibility exercises included a static stretching routine of major upper- and lower-body muscle groups. At 4 months, use of gym-based equipment (hand and ankle weights, resistance tubing, and balance foams) was integrated into the strength and balance exercises.

Training was progressive, with challenges increasing with respect to movement pace, patterns and coordination, and joint range of motion. Strength training was graduated, beginning with 4 repetitions in month 1, 6 to 8 repetitions in month 2, 8 to 10 repetitions in month 3, 11 to 15 repetitions in month 4, and 25 to 30 repetitions in months 5 and 6. Resistance training involved hand weights (beginning with 0.45 kg [1 lb] for each hand in month 4 and progressing to 0.91 kg [2 lb] in months 5 and 6), tubing (beginning with extra-light resistance in month 4, moving to light resistance in month 5, and to medium resistance in month 6), and ankle weights (beginning with 0.45 kg for each limb in month 4 and progressing to 1.13 kg [2.5 lb] in month 6). These resistance exercises were implemented with 3 to 5 repetitions in month 4, increasing to a maximum of 8 to 10 repetitions in month 6.

#### Stretching Exercise

The training routine consisted of breathing, stretching, and relaxation activities, with most of them performed in a seated position. Each session began with a set of warm-up exercises, such as arm, neck, and leg circles; trunk rotation; and light walking. The core part of the training session consisted of a variety of combined seated and standing stretches involving the upper body (neck, arms, upper back, shoulders, and back and chest) and lower extremities (quadriceps, hamstrings, calves, and hips), along with slow and gentle trunk rotations. Also included were deep abdominal breathing exercises that emphasized inhaling and exhaling to maximum capacity as well as progressive relaxation of major muscle groups.

### Baseline and Outcome Assessment

At enrollment, participants’ demographic information regarding sex, age, race/ethnicity, income, education, living arrangements, medical conditions, fall-related information, and physical activity was collected. Study outcome measures were assessed at baseline, 4 months (midpoint), and 6 months (at the end of the intervention).

The primary outcome was the incidence of falls, which was ascertained on a monthly basis. Participants were asked to use a daily “fall calendar”^13^ diary to record any fall event (defined as “when you land on the floor or the ground, or fall and hit objects like stairs or pieces of furniture, by accident”) and to indicate whether they sought medical attention. Information was also collected on injurious falls.^13,22^ Data on falls were collected starting from the date of the first intervention class and continuing until 24 weeks later (ie, the end of the intervention period) or until a participant withdrew, died, or was lost to follow-up.

Prespecified secondary outcomes were physical performance measures of (1) functional reach,^23^ which assessed the maximal distance a participant could reach forward, beyond arm’s length, while maintaining a fixed based of support in a standing position; (2) the Instrumented Timed Up & Go (APDM, Inc), which represents an extended version of TUG^18^ and assessed walking duration (in seconds) and 3 subdomain timed-based activities—sit-to-stand, turning, and turn and stand-to-sit—during a 14-m walk at normal pace (7 m toward a line, turn, and 7 m toward the chair); and (3) the Short Physical Performance Battery,^24^ which measured repeated chair stands, 3 increasingly challenging standing balance tasks, and a 4-m speed walk. Scores on the 3 tasks were combined to create an overall performance score of 0 (worst) to 12 (best), with higher values indicating improvement. In addition, global cognitive function was measured by the 30-item Montreal Cognitive Assessment,^25^ which assesses cognitive function of multiple domains (memory recall, visuospatial abilities, executive functions, attention, language, and orientation to time and place; scores range from 0 to 30, with higher scores indicating better cognitive function).

### Statistical Analysis

#### Sample Size

The study was powered to detect a difference between 2 negative binomial rates resulting from the 6-month intervention between the 2 exercise interventions (TJQMBB and multimodal exercise) relative to stretching exercise. On the basis of data collected from previous trials,^13,16^ our power calculations found that a sample size of 567 participants (189 per group) would be required to detect a 35% reduction in the fall incidence rate (a respective incidence rate ratio [IRR] of 0.65) between either of the 2 intervention groups relative to stretching exercise. Although a difference was anticipated to favor the TJQMBB intervention, power was not calculated between TJQMBB and multimodal exercise owing to the lack of a priori effect size estimates. With an estimated 15% attrition, we planned to recruit a total of 666 participants.

#### Analyses

Baseline characteristics and unadjusted study outcome measures were summarized by intervention group using descriptive statistics such as mean (SD) or percentage and used to assess between-group equivalence at baseline. Prespecified baseline covariates in both primary and secondary outcome analyses included age, sex, health status, history of falls, and cognitive function (Mini-Mental State Examination score, ≤20).

Baseline demographic descriptors and primary and secondary outcome measures were compared across groups by using analysis of variance for continuous variables and the χ^2^ (or Fisher exact) test for categorical variables. The planned descriptive data on monthly falls was tabulated across the intervention groups. In our primary analysis of the falls count outcome, we used negative binomial regression to estimate absolute differences in IRRs with their corresponding 95% CIs comparing TJQMBB and multimodal exercise with stretching exercise. In a prespecified secondary analysis, we also estimated the rate differences with 95% CIs between TJQMBB and multimodal exercise. Follow-up on falls data was censored at the last visit or contact during which a complete data point was collected. Following an intention-to-treat protocol, we analyzed the secondary (continuous) outcomes with estimates and their 95% CIs generated from the linear mixed-effects models. All primary and secondary outcome analyses were conducted with and without adjustment for prespecified baseline covariates. Bonferroni correction was made to control for multiple testing of secondary outcomes, with an adjusted α value of .007 (.05 per 7 comparisons) for each test considered statistically significant. Two-sided *P* values of less than .05 were considered statistically significant. Analyses were conducted using SPSS version 23 (IBM Corp) or Stata (release 13; StataCorp LP).

## Results

### Enrollment

Of 1147 individuals screened, 670 were enrolled and randomized (224 to TJQMBB, 223 to multimodal exercise, and 223 to stretching exercise) (Figure). Of the total participants, 581 (86.7%) (194 in TJQMBB, 193 in multimodal exercise, and 194 in stretching exercise) completed their assigned interventions. At 6 months, 664 (99.1%) of the 670 participants provided full follow-up data on falls and 633 (94.5%) provided data on secondary outcomes. There were no statistically significant differences in baseline demographic variables or primary outcomes between the 581 participants who completed the intervention (defined as attending classes either regularly or irregularly without dropping out of the study) and the 89 who did not complete the intervention (defined as dropping out of the study).

### Participant Characteristics

Baseline characteristics were similar by intervention group (Table 1). The mean (SD) age was 77.7 (5.6) years (median [interquartile range (IQR)], 76 [73-81] years), 436 (65.1%) were women, 617 (92.1%) were white, 31 (4.6%) were African American. Four hundred eighty-five participants (72.4%) reported having at least 1 fall 6 months before the intervention, 355 (53.0%) reported having 3 or more chronic conditions, and 67 (10.0%) were taking 4 or more medications. The mean (SD) mobility score was 8.3 (2.2) measured on the Short Physical Performance Battery and 14.25 (5.2) seconds on TUG.

**Table 1.** Baseline Demographic and Clinical Characteristics of the Study Population

Characteristic	TJQMBB (n = 224)	Multimodal Exercise (n = 223)	Stretching Exercise (n = 223)
Age, mean (SD), y	77.5 (5.6)	77.8 (5.3)	77.8 (5.9)
Sex, No. (%)			
Male	78 (34.8)	80 (35.9)	76 (34.1)
Female	146 (65.2)	143 (64.1)	147 (65.9)
Race/ethnicity, No. (%)			
White	203 (90.6)	203 (91.0)	211 (94.6)
African American	13 (5.8)	14 (6.3)	4 (1.8)
Other	8 (3.6)	6 (2.7)	8 (3.6)
Educational level, No. (%)			
High school diploma or lower	95 (42.4)	101 (45.3)	92 (41.3)
College degree or higher	129 (57.6)	122 (54.7)	131 (58.7)
Resting blood pressure, mean (SD), mm Hg			
Systolic	133.3 (19.3)	135 (19.7)	134.1 (19.1)
Diastolic	75.6 (11.3)	75.6 (10.5)	74.2 (11.5)
Body mass index, mean (SD)^a^	29.2 (6.0)	29.4 (6.6)	29.91 (6.2)
Self-reported fear of falling, No. (%)			
No fear	23 (10.3)	22 (9.9)	32 (14.4)
Somewhat	132 (58.9)	129 (57.8)	116 (52.0)
A lot to very much	69 (30.8)	72 (32.3)	75 (33.6)
Self-reported falls in previous 6 mo, No. (%)			
None	63 (28.1)	61 (27.4)	61 (27.4)
1	79 (35.3)	71 (31.8)	72 (32.3)
2	46 (20.5)	56 (25.1)	48 (21.5)
≥3	36 (16.1)	35 (15.7)	42 (18.8)
Self-reported health status, No. (%)			
Poor or very poor	44 (19.6)	40 (17.9)	46 (20.6)
Good or fair	113 (50.5)	117 (52.5)	106 (47.6)
Very good or excellent	67 (29.9)	66 (29.6)	71 (31.8)
Self-reported chronic conditions, No. (%)			
None	11 (4.9)	12 (5.4)	9 (4.0)
1	32 (14.3)	33 (14.8)	40 (17.9)
2	60 (26.8)	63 (28.2)	52 (23.4)
≥3	121 (54.0)	115 (51.6)	122 (54.7)
Self-reported medication use, No. (%)			
None	41 (18.3)	47 (21.1)	51 (22.9)
1 Medication	59 (26.3)	66 (29.6)	61 (27.4)
2 Medications	67 (29.9)	60 (26.9)	57 (25.5)
≥3 Medications	57 (25.5)	50 (22.4)	54 (24.2)

Abbreviation: TJQMBB, *Tai Ji Quan*: Moving for Better Balance.

^a^ Calculated as weight in kilograms divided by height in meters squared.

### Intervention Compliance and Adherence

The overall attrition rate was 13%, which was lower than our planned 15%. The median (IQR) time to stopping the intervention across the 3 intervention groups was 3.0 months (2.0-4.0). The intervention attendance rate across the 24-week period for all participants was 77% (78% in the TJQMBB group; 77% in the multimodal exercise group; and 77% in the stretching exercise group), and the mean (SD) number of completed sessions was 37 (10.6) (median, 40 sessions; range, 2-48 sessions) (37 [10.8] in the TJQMBB group; 37 [10.2] in the multimodal exercise group; and 37 [10.7] in the stretching exercise group; *P* = .82).

### Safety

Serious adverse events, defined as death or medical conditions that required 1 or more days of hospitalization, were observed. Forty-seven participants reported hospital admission: 11 in the TJQMBB group (1.0 per 100 person-months), 12 in the multimodal exercise group (1.1 per 100 person-months), and 16 in the stretching exercise group (1.2 per 100 person-months) (*P* = .83). One death from unknown causes in the stretching exercise group was documented. None of these serious events were related to the intervention. Seven falls with no injury were documented during classes: 2 in the TJQMBB group; 3 in the multimodal exercise group; and 2 in the stretching exercise group. One participant in the TJQMBB group required an emergency department visit owing to hyponatremia during a class, but the participant recovered and completed the intervention.

### Intervention Resource Use

The resources needed to conduct our intervention primarily involved costs associated with promotion, recruitment, room rental, class instruction, supplies, administrative overhead, exercise equipment (eg, weights, chairs), and participant travel expenses to and from each intervention class. The intervention cost $202 949 ($906 per person) to deliver a 24-week TJQMBB program to 224 participants, $223 849 ($1004 per person) to deliver the multimodal exercise program to 223 participants, and $201 468 ($903 per person) to deliver the stretching exercise component to 223 participants.

### Primary Outcome

At 6 months, 733 falls were recorded among 324 of the 670 participants (48.4%) (85 in the TJQMBB group, 112 in the multimodal exercise group, and 127 in the stretching exercise group ). The mean (SD) follow-up on falls was 5.98 (0.21) months (median [IQR], 6.0 [6.0-6.0] months). Although both the TJQMBB and multimodal exercise groups showed a significantly lower incidence of falls (11 per 100 person-months for TJQMBB, and 16 per 100 person-months for multimodal exercise) compared with the stretching exercise group (27 per 100 person-months, *P* < .001), the incidence of falls was significantly lower in the TJQMBB group (total falls [mean (SD)] 152 [0.68 (1.3)]) than in the multimodal exercise group (218 [0.98 (1.8)]) (*P* = .04). There were no between-group differences on moderate injurious falls (TJQMBB, 88 falls [0.39 (0.9)]; multimodal exercise, 109 [0.49 (1.2)]; and stretching exercise, 156 [0.70 (1.7)]) (*P* = .05), but TJQMBB had a lower incidence of injurious falls than stretching exercise (TJQMBB, 8 [0.04 (0.2)]; stretching, 25 [0.11 (0.4)]) (*P* = .008) (Table 2).

**Table 2.** Incidence of Falls During the 24-Week Intervention by Intervention Group

Falls^a^	TJQMBB (n = 224)	Multimodal Exercise (n = 223)	Stretching Exercise (n = 223)
Total falls, No. (mean) [SD]	152 (0.68) [1.3]	218 (0.98) [1.8]	363 (1.63) [3.9]
No. of falls, No. (%) of participants			
Any	85 (37.9)	112 (50)	127 (57)
1	55 (24.6)	68 (30.5)	62 (27.8)
2	13 (5.8)	25 (11.2)	31 (13.9)
≥3	17 (7.6)	19 (8.5)	34 (15.2)
Total injurious falls, No. (mean) [SD]			
Moderate^b^	88 (0.39) [0.9]	109 (0.49) [1.2]	156 (0.70) [1.7]
Serious^c^	8 (0.04) [0.19]	14 (0.06) [0.26]	25 (0.11) [0.37]

Abbreviation: TJQMBB, *Tai Ji Quan*: Moving for Better Balance.

^a^ Data are based on all available participants who provided fall data during the 24-week intervention period.

^b^ Moderate injurious falls are defined as those that resulted in sprains, bruises, scrapes, or joint injuries but that required no professional medical care. None of these injuries was related to the intervention. There were no between-group differences on moderate injurious falls (*P* = .05).

^c^ Serious injurious falls are defined as those that resulted in a fracture, head injury, or admission to an emergency department or hospital, or that required stitches. None of these injuries was related to the intervention. The TJQMBB group had a lower incidence of serious injurious falls compared with the stretching exercise group (*P* = .008).

Binominal regression of unadjusted analyses showed that both the TJQMBB and multimodal exercise groups had a lower IRR (IRR, 0.42; 95% CI, 0.31-0.56; *P* < .001 for TJQMBB; IRR, 0.60; 95% CI, 0.45-0.80; *P* = .001 for multimodal exercise) compared with the stretching exercise group. In addition, the TJQMBB group showed a significantly lower IRR than the multimodal exercise group (IRR, 0.69; 95% CI, 0.52-0.94; *P* = .01). The estimates of the intervention effects between TJQMBB and the stretching and multimodal exercise groups showed no change after adjusting for the prespecified covariates (data not shown).

### Secondary Outcomes

At 6 months, the participants in both the TJQMBB and multimodal exercise groups performed significantly better than those in the stretching exercise group on secondary outcomes of physical performance (functional reach, Short Physical Performance Battery, and Instrumented TUG and its subdomain scores [sit-to-stand, turning, turn and stand-to-sit]) and global cognitive function measures (Table 3). Participants in the TJQMBB and multimodal exercise interventions performed significantly better than those in the stretching intervention on tests of physical and cognitive function: functional reach (TJQMBB: mean difference, 1.77; 95% CI, 1.42-2.12; *P* < .001; multimodal exercise: mean difference, 1.49; 95% CI, 1.15-1.83; *P* < .001); Short Physical Performance Battery (TJQMBB: mean difference, 1.57; 95% CI, 1.25-1.88; *P* < .001; multimodal exercise: mean difference, 1.59; 95% CI, 1.27-1.90; *P* < .001); total walking duration in the instrumented walking test (TJQMBB: mean difference, −2.42; 95% CI, −3.19 to −1.65, *P* < .001; multimodal exercise: mean difference, −2.20; 95% CI, −2.97 to −1.43; *P* < .001); and Montreal Cognitive Assessment (TJQMBB group: mean difference, 1.54; 95% CI, 1.04-2.04; *P* < .001; multimodal exercise group: mean difference, 1.39; 95% CI, 0.92-1.86; *P* < .001). There were no differences between TJQMBB and multimodal exercise on secondary outcomes. The significant effects of TJQMBB and multimodal exercise relative to stretching exercise on the secondary outcomes remained after adjustment for covariates (data not shown).

**Table 3.** Secondary Outcomes at Baseline, 4 Months, and 6 Months and Group Differences in Change

Outcome	TJQMBB (n = 224)	Multimodal Exercise (n = 223)	Stretching Exercise (n = 223)	Between-Group Difference in Mean Changes From Baseline (95% CI)		
				TJQMBB vs Stretching Exercise	TJQMBB vs Multimodal Exercise	Multimodal Exercise vs Stretching Exercise
Functional reach, mean (SD), in						
Baseline	8.09 (2.34)	8.14 (2.09)	8.11 (2.53)	1.77 (1.42 to 2.12)	0.28 (−0.04 to 0.60)	1.49 (1.15 to 1.83)
4 mo	8.76 (2.62)	8.73 (2.64)	8.38 (2.67)			
6 mo	10.07 (2.26)	9.84 (2.32)	8.32 (2.65)			
SPPB score, mean (SD)						
Baseline	8.09 (2.23)	8.12 (2.04)	8.22 (2.15)	1.57 (1.25 to 1.88)	−0.02 (−0.35 to 0.30)	1.59 (1.27 to 1.90)
4 mo	8.68 (2.29)	9.06 (2.16)	8.48 (2.44)			
6 mo	9.83 (1.85)	9.87 (1.85)	8.39 (2.52)			
iTUG mean (SD), s^a^						
Total walking duration						
Baseline	22.96 (5.94)	22.78 (6.05)	22.76 (6.00)	−2.42 (−3.19 to −1.65)	−0.22 (−0.83 to 0.39)	−2.20 (−2.97 to −1.43)
4 mo	22.18 (5.83)	21.75 (6.13)	22.58 (6.83)			
6 mo	20.86 (5.13)	20.89 (5.92)	23.09 (7.89)			
Sit-to-stand duration						
Baseline	2.44 (0.44)	2.43 (0.40)	2.39 (0.45)	−0.34 (−0.44 to −0.23)	−0.04 (−0.12 to 0.05)	−0.30 (−0.41 to −0.19)
4 mo	2.39 (0.45)	2.42 (0.40)	2.33 (0.46)			
6 mo	2.12 (0.36)	2.14 (0.33)	2.41 (0.62)			
Turning duration						
Baseline	2.92 (0.75)	2.91 (0.78)	2.91 (0.85)	−0.48 (−0.62 to −0.34)	−0.11 (−0.23 to 0.00)	−0.37 (−0.50 to −0.23)
4 mo	2.76 (0.75)	2.72 (0.82)	2.88 (1.10)			
6 mo	2.43 (0.59)	2.53 (0.77)	2.90 (1.18)			
Turn and stand-to-sit duration						
Baseline	4.85 (1.10)	4.85 (1.08)	4.85 (1.68)	−0.67 (−0.92 to −0.42)	−0.03 (−0.17 to 0.12)	−0.64 (−0.89 to −0.40)
4 mo	4.66 (1.06)	4.59 (1.22)	4.87 (2.03)			
6 mo	4.30 (0.98)	4.34 (0.90)	4.98 (2.56)			
MoCA score, mean (SD)						
Baseline	24.70 (3.02)	24.75 (3.20)	24.78 (3.24)	1.54 (1.04 to 2.04)	0.15 (−0.33 to 0.64)	1.39 (0.92 to 1.86)
4 mo	25.37 (3.64)	25.32 (3.13)	24.96 (3.34)			
6 mo	26.33 (3.54)	26.23 (2.95)	24.88 (3.50)			

Abbreviations: iTUG, Instrumented Timed Up & Go; MoCA, Montreal Cognitive Assessment; SPPB, Short Physical Performance Battery; TJQMBB, *Tai Ji Quan*: Moving for Better Balance.

^a^ The test involves standing up from a chair, walking 7 m, turning around, walking back (7 m), and sitting down in the chair.

## Discussion

In this study of community-dwelling older adults at high risk of falling, we found that a 6-month TJQMBB intervention, when compared with a conventional stretching exercise control, was effective in reducing the incidence of falls. In addition, our study also showed for the first time, to our knowledge, that TJQMBB was effective in reducing the incidence of falls compared with a well-known conventional, evidence-based multimodal exercise program.^9,16^ Thus, of the 3 exercise interventions, TJQMBB yielded the greatest reduction in number of falls, whereas both TJQMBB and multimodal exercise significantly improved physical function and global cognitive function compared with the stretching exercise control.

The findings from this study are aligned with systematic review and meta-analyses on the effect of exercise on reducing the incidence of falls^7,26^ and are commensurate with the results from a meta-analysis^27^ and previous controlled *tai ji quan* studies involving community-dwelling older adults^13,28,29^ and persons with Parkinson disease.^30^ This clinically oriented and functionally driven *tai ji quan*–based program,^21^ however, is shown to be more efficacious in the magnitude of reduction in the incidence of falls compared with earlier trial or meta-analysis results.^13,27,28,29^

Our study also extends the current literature by comparing, head-to-head, 2 evidence-based interventions,^13,16^ with the results showing 31% fewer falls in TJQMBB compared with multimodal exercise, thus adding new clinical knowledge on the effectiveness of a therapeutically tailored *tai ji quan* intervention strategy for preventing falls among older adults. The findings that TJQMBB was more effective than a multimodal exercise program are of considerable practical importance because they suggest the utility of an equipment-free, low-cost, non–space-constrained exercise intervention in addressing the clinical problem of falls and balance deficits in the older population.

To our knowledge, our intervention is the only *tai ji quan*–based program uniquely designed to facilitate therapeutic training of balance and postural control for older adults with balance deficits,^14,15,21^ with the specific focus of targeting reductions in falls and tailoring implementation for clinical practice. A previous study has shown that the intervention is readily implementable in clinical practice with a high rate of adoption among health care practitioners, including internal medicine physicians, and that it is sustainable.^14^ Experience from that study indicated that proactive steps, such as communicating frequently with clinicians, offering educational outreach workshops, and even providing training to clinicians, can facilitate the referral process. Thus, although substantial communication gaps exist between clinicians and community service providers,^9^ we have shown that this program can be accessible to clinicians and implementable in the context of geriatric clinics or medical centers.

The TJQMBB intervention evaluated in this study has been the model program for multiple successful research-to-practice implementation efforts in both community and clinical settings as well as program delivery evaluation by public health organizations or senior service agencies by public health organizations or senior service agencies.^14,15,31,32,33,34,35^ The program is also currently listed as one of the highest-tier evidence-based health-promoting and disease prevention programs under Title IIID of the Older Americans Act.^36^ With increasing evidence of community adoption and implementation^14,15,31,32,33,34,35^ and information from cost-benefit and cost-effectiveness analyses,^15,37^ the intervention program represents a promising approach to low-cost and easily implementable fall prevention programs. Its demonstrated generalizability and scalability can facilitate nationwide adoption of this effective fall prevention program to benefit community-dwelling older adults.

### Limitations

The study findings should be interpreted in the context of trial limitations. Falls data were collected via falls calendars kept by participants. Although such calendars remain the criterion standard for ascertaining best available evidence on falls in the field,^13,16,22,38^ self-reports are known to be subject to recall bias. However, to ensure that self-reporting bias was minimized, we used multiple methods, including monthly telephone calls, confirmations during follow-up assessments, proxies, and medical records, to ensure data accuracy. Participation in the study classes required traveling. Therefore, the results are most likely to be generalizable to persons who are able to travel regularly to exercise class sites. The relatively low representation of African American participants was noted given this group has high rates of falls and of injurious falls.^2^ However, there is no indication in the results that these participants responded differently to the interventions than did other participants. Finally, this trial was conducted in a single state. Although Oregon has one of the nation’s highest death rates from falls,^39^ generalizability of the findings could be enhanced by a multicenter trial involving multiple states.

## Conclusions

Among older adults with high risk of falling, a 24-week therapeutically developed *tai ji quan* balance training intervention resulted in a significant reduction in the incidence of falls compared with a stretching exercise modality and a multicomponent exercise program.

## Notes

Supplement. Trial Protocol
