# Supplementary material for: Effectiveness of a Therapeutic Tai Ji Quan Intervention vs a Multimodal Exercise Intervention to Prevent Falls Among Older Adults at High Risk of Falling: A Randomized Clinical Trial
Source: JAMA Intern Med. 2018 Sep 10;178(10):1301–10. doi: 10.1001/jamainternmed.2018.3915 (PMC6233748; doi:10.1001/jamainternmed.2018.3915)
Supplement: Supplement. — Trial Protocol [file jamainternmed-178-1301-s001.pdf]

Supplement

Trial Protocol

Information described in this protocol formed part of the original submission.

Supplement to the paper by: Li F, Harmer P, Fitzgerald K, Eckstrom E, Akers L, Chou L-S, Pidgeon D, Voit J, Winters-Stone K. Effectiveness of a therapeutic *tai ji quan* intervention vs a multimodal exercise intervention to prevent falls among older adults at high risk of falling: a randomized clinical trial.

Trial Protocol

Study Aims

The objective of this study was to conduct a comparative effectiveness study of a therapeutically tailored, evidence-based fall prevention program—*Tai Ji Quan: Moving for Better Balance* (TJQMBB) versus an evidence-based multimodal exercise intervention in reducing the number of falls among older adults at high risk of falling.

Study Hypotheses

Our primary hypothesis was that TJQMBB would be clinically more effective in reducing the number of falls compared with Multimodal or Stretching interventions. In addition, we predicted that, relative to the Stretching control, both TJQMBB and Multimodal Exercise would result in improvements in secondary outcome measures of physical performance and global cognitive function.

Study Design

The study used a single-blind, parallel design, randomized controlled trial with participants randomly allocated to one of three active arms: TJQMBB, entailing modified Tai Ji Quan forms and therapeutic movement exercises; Multimodal, integrating aerobic, strength, balance, and flexibility activities; or Stretching exercise, serving as minimal practice standard (the control arm).

Trial Protocol Approval

The trial protocol was approved by the Institutional Review Board of Oregon Research Institute. An independent Data and Safety Monitoring Board appointed by the National Institute on Aging oversaw the study. Informed consent was obtained from all participants.

Trial and Protocol Registration

The study and its protocol were registered on Clinicaltrials.gov (NCT02287740).

Study Population

Target prospects were community-dwelling older adults living in urban and suburban cities (Eugene, Springfield, Portland metro area) in Oregon.

Eligible participants were 70 years of age and older who met one of the following primary criteria: (a) having had at least 1 fall in the preceding 12 months and having a healthcare provider's referral indicating that the participant was at risk of falls, or (b) having impaired mobility as evidenced by a Timed Up and Go[1] result of >13.5 seconds.[2] Other inclusion criteria were: (a) being able to walk 1 or 2 blocks, with or without the use of an assistive device, (b) being able to exercise safely as determined by a healthcare provider, and (c) having a willingness to be randomly assigned to an intervention condition and complete the 6-month intervention. Individuals were excluded if they (a) were participating in daily and/or structured vigorous physical activity or walking for exercise that lasted 15 minutes or longer or muscle-strengthening activities (e.g., weight lifting) on 2 or more days a week in the previous 3 months, (b) had severe cognitive impairment (Mini-Mental State Examination [MMSE][3] score of 20 or less), and (c) had medical or physical conditions determined by their healthcare provider to preclude exercise.

Recruitment Sources and Procedures

Recruitment strategies included promotions at local senior/community centers, senior meal sites, healthcare professional clinics and medical clinics, statewide senior falls prevention networks, targeted mass mailings, and local newspaper advertisements. To reduce potential expectation bias, participants were informed that the study would be comparing three different exercise interventions and that they would be assigned to an exercise group at random.

To ascertain eligibility, a research assistant made an initial telephone contact with those who responded to the study promotion. This initial prescreening telephone contact was intended to determine

basic eligibility set forth under the recruitment inclusion and exclusion criteria. Potentially eligible participants who met the initial inclusion criteria were scheduled for a 2-hour in-office visit at one of our research offices.

Baseline Assessments

Those individuals who were eligible after screening were then given informed consent materials followed by a baseline evaluation conducted by a study assessor. The baseline assessments included blood pressure, weight and height, and collection of information on demographics, health status, and current medical conditions and medication use. This was followed by assessments of falls, mobility, physical performance, and cognitive ability. At that time, the intervention activities involved in the study were reiterated to the eligible individuals. Those who met all study criteria, were clear about research procedures, had completed baseline assessments, and gave final consent were then randomized.

Randomization and Blinding

Eligible participants were randomized to one of the interventions with an allocation of 1:1:1, through a permuted block randomization. Concealment of allocation was implemented. The randomization schedule, generated by the project data analyst, was kept by a project staff member who delivered it, in a sealed envelope, to a research assistant who then assigned qualified individuals to intervention groups. Randomization occurred after informed consent was obtained and baseline assessments were completed.

All study assessors who collected study outcome measures were blinded to study hypotheses and group allocation. Blinding was strictly maintained by emphasizing to assessors the importance of minimizing assessment bias and regular checking of the blinding status. Efforts were also made to maintain separation between the study assessors and research assistants who dealt with administrative activities and class safety monitoring, and between study assessors and class instructors who delivered the intervention. Participants were instructed not to reveal their group status to the study assessors at any time. The investigators and the data analyst were blinded to group designation and reviewed coded data with names of participants replaced by numbers.

Intervention

Participants in each intervention group participated in twice weekly training sessions, 60 minutes in length, for 24 weeks. In all three groups, each session consisted of (a) 10-15 minutes of warm-up, (b) 40-45 minutes of core exercises, and (c) 5 minutes of cool-down activities involving muscle relaxation and breathing exercises. No additional in-home or between-sessions exercises were assigned for any of the three interventions during the study period. To ensure consistency in delivery, this protocol remained the same throughout the 24 weeks of training.

TJQMBB. The protocol involved a core 8-form routine with built-in practice variations and a subroutine of integrated therapeutic movements.[4] Aimed at stimulating and integrating musculoskeletal, sensory, and cognitive systems, core practice employed controlled, self-initiated, Tai Ji Quan-based movements focused on the center of body mass with synchronized breathing. Unlike conventional Tai Ji Quan practice, all exercises in TJQMBB were executed through a dynamic interplay of stabilizing and destabilizing postural actions with movements involving unilateral weight-bearing and weight-shifting, trunk and pelvic rotation, ankle sways, and eye-head-hand movements.

During the initial 10 weeks, training primarily involved learning and practicing forms arranged in various formats (i.e., seated, standing in place, stepping), accompanied by sets of mini-therapeutic movements that involved ankle sway, sit to stand, single-leg stand, turning, and stepping exercises. In each session, there were 3-4 sets (with 3-5 repetitions in each set) of each practice format intermingled with 3-5 sets (with 4-5 repetitions in each set) of 3-4 selected mini-therapeutic movements. After all 8 forms were taught (around Weeks 11-12), training at each session was maintained at between 5-6 sets of variations in the 8-form routine with a minimum of 3-4 mini-therapeutic movements being practiced in sets (4-5). The instructional guide for teaching is available at tjqmbb.org (accessible to trained and

registered TJQMBB instructors only). Instructional progression of exercise activities and intensity in the TJQMBB protocol was standardized to the group but modified for participants who had physical limitations or had missed multiple class sessions.

Multimodal Exercise. Developed by Lord et al.[5] and recommended by the CDC,[6] this is a tailored, progressive, multimodal program that involves aerobic, strength, balance, and flexibility exercises. The aerobic exercises included long strides, heel-toe walking, narrow- and wide-based walking, and sidestepping. Strength training included exercises for ankle dorsiflexors, knee extensors, and hip abductors. Balance training involved tandem foot-standing, heel-toe and line walking, single-leg standing, alternation of the base of support, weight transfers, toe and heel movements, and various reaching and stretching movements away from the center of gravity. Flexibility exercises, performed in both seated and standing positions, included a static stretching routine of major upper and lower body muscle groups. At 4 months, use of gym-based equipment such as hand and ankle weights, resistance tubing, and balance foams was integrated into the strength and balance exercises.

Training was progressive, with increasing challenges with respect to movement pace, patterns and coordination, and joint range of motion. Strength training was graduated, beginning with 4 repetitions in Month 1, 6-8 repetitions in Month 2, 8-10 repetitions in Month 3, 11-15 repetitions in Month 4, and 25-30 repetitions in Months 5-6. Resistance training involved hand weights (beginning with 0.45 kg for each hand in Month 4 and progressing to 0.91 kg in Months 5 and 6); tubing (beginning with extra-light resistance in Month 4, moving to light in Month 5, and to medium resistance in Month 6); and ankle weights (beginning with 0.45 kg for each limb in Month 4 and progressing to 1.13 kg in Month 6). These resistance exercises were implemented with 3-5 repetitions in Month 4, increasing to a maximum of 8-10 repetitions in Month 6. Similar to TJQMBB, progression of exercise activities and intensity in the protocol was standardized to the group but modified for participants who had physical limitations or had missed multiple class sessions.

Stretching exercise. The rationale for using this modality was to provide participants with a low-intensity exercise program that would contain identical social interaction, enjoyment, and physical activity (i.e., by traveling to and from the training centers) and changes in lifestyle secondary to study participation that were inherent components in the other two exercise interventions without providing comparable lower-extremity weight-bearing strength or balance training benefits. The validity of this program has been demonstrated in a previous study, which showed no significant effects on physical performance measures.[7] The program consisted of breathing, stretching, and relaxation, with the majority of activities performed in a seated position. Each session began with a set of warm-up exercises such as stretching arms, neck, shoulder and back, hips, thighs, and legs; ankle and trunk rotation; and light walking. The core part of the training session consisted of exercises that encompassed a variety of seated-and-standing combined stretches involving the upper body (arms, neck, upper back, shoulder, back, and chest), lower extremities (quadriceps, hamstrings/calfs, and hips), and gentle and slow trunk rotations. Also included were deep abdominal breathing exercises that emphasized inhaling and exhaling to maximum capacity, as well as progressive relaxation of major muscle groups.

Exercise Intensity

Practice intensity level, assessed through a subjective measure of perceived exertion, was closely monitored throughout the 24-week study period for all three active exercise interventions. Specifically, during the first 10 weeks, participants in TJQMBB and Multimodal Exercise were instructed to exercise at a level of exertion that they characterized as being "Light to Moderate" (equivalent to 2-4 on the 0-10 Borg CR10 scale) and progressed to "Moderate to Strong" and "Strong" (equivalent to 5-6 on the 0-10 scale) after 10 weeks of intervention. Exercise intensity in the Stretching control was kept between "Very Light" (1) and "Light" (2) throughout the 24-week intervention period.

Class Size

A class size of 8-15 was planned in order to provide adequate instructional attention to each participant and to allow the class instructor to carry out the required training routines.

Class Location

Intervention classes were offered at sites where aging services were available, including community health centers, medical health centers, independent-living residential communities, non-profit organizations, and faith-based organizations, and where evidence-based disease prevention and health promotion activities were encouraged.[8]

Class Instructors

Class instructor training for study protocols was standardized and equivalent with respect to the time spent on training orientation across interventions. TJQMBB instructors were trained by the first author prior to intervention, and the instructors who taught Multimodal and Stretching were community instructors with a background in exercise science and equivalence in teaching experience. There was no cross-over teaching among the TJQMBB, Multimodal, and Stretching groups.

Drop-outs

Unavoidable drop-outs, from causes such as death, onset of severe illness, or other medical complications, were anticipated. Based on our prior studies, we estimated a 15% dropout rate for the overall study. The proposed sample size took into account this anticipated dropout rate.

Program Fidelity

A standardized intervention protocol and a process evaluation checklist, developed via prior trials,[7,9] was implemented. These measures focused primarily on intervention fidelity and involved issues such as (a) instructor qualification and training, (b) teaching distribution of the individual forms or movements, (c) exercise intensity and consistency in training dosage across different sites, and (d) weekly class attendance checking and monitoring. The evaluation was conducted monthly by authorized personnel.

Data Collection and Outcome Measurements

All assessments were conducted at the research laboratories of the Oregon Research Institute in Eugene and Portland. At baseline, demographic and individual profile measures included sex, age, ethnicity, income, education, medical conditions, fall-related information, and physical activity.[10] Outcome measures were assessed at baseline, 4 months (the beginning of the fourth month), 6 months (intervention termination), and again at 12 months (post-intervention follow-up).

The primary endpoint was all falls reported by participants across the 24-week surveillance period. Using a daily diary "falls calendar,"[7,9] participants were asked to record any fall event (defined as "when you land on the floor or the ground, or fall and hit objects like stairs or pieces of furniture, by accident") and to indicate whether the fall caused them to seek medical attention. A fall was considered to have caused (1) "moderate injury" if the fall resulted in sprains, bruises, scrapes, or joint injuries or if the individual sought medical care, and (2) "serious injury" if the fall resulted in a fracture, head injuries, or admission to a hospital or resulting in an injury requiring stiches.[7,9,11,12] Blinded assessors verified injury falls from medical records.

Ascertainment. Multiple approaches were used to ascertain data on falls. These included the following:

1. monthly collection of falls calendars
2. monthly phone verification by a blinded research assessor
3. self-report from each follow-up assessment (4 months, 6 months, or information immediately reported by the participant or his/her spouse)

Secondary outcomes included (a) Functional Reach,[13] assessing the maximal distance one can reach forward and backward, beyond arm's length, while maintaining a fixed based of support in the standing position; (b) 7-meter Instrumented Timed Up and Go (I-TUG; APDM, Inc.),[14,15] assessing four time-based parameters involving (1) stride velocity, (2) sit-to-stand, (3) turning, and (4) turn and stand-to-

sit, during a 14-meter walk at normal pace (7 meters towards a line, turn, and 7 meters towards the chair); (c) Short Physical Performance Battery,[16,17] measuring (1) repeated chair stands, (2) three increasingly challenging standing balance tasks, and (3) a 4-meter walk; and (d) a cognitive measure (the Montreal Cognitive Assessment),[18] assessing global cognitive function in multiple domains (memory recall, visuospatial abilities, executive functions, attention, language, and orientation).

Statistical Analysis

Baseline characteristics were summarized by intervention group using mean and standard deviation, or percentages. Intervention adherence was calculated as the percentage of scheduled intervention sessions attended by participants. Data on monthly falls frequency was tabulated across the intervention groups.

Monthly falls data collected across the entire 24-week intervention period was analyzed to test our primary hypothesis. To compare fall incident rates between intervention groups, we used negative binomial regression models, which generated incidence rate ratios (IRRs) with the corresponding 95% confidence intervals (CIs). Follow-up was censored at the last visit where the complete data point was collected.

In our negative binomial regression analysis, using Stretching as a reference, we compared the fall rates between TJQMBB and Stretching and between Multimodal Exercise and Stretching. In a pre-specified analysis, we also compared the effect between TJQMBB and Multimodal Exercise. We ran our end point analysis twice: one without any covariates (specified below) and one with all important covariates. In the latter analysis, we adjusted or controlled for important baseline covariates including age (70-74, ≥75), sex, number of falls in previous 3 months at baseline, drug use (taking more than four drugs at baseline), number of chronic conditions, MMSE, and physical activity. No pre-specified subgroup analyses were conducted.

In the primary analysis on fall rates between groups, two-sided P values of less than .05 were considered statistically significant. No Bonferroni correction was applied.

The intervention effects on secondary outcomes were analyzed using mixed-effects models for repeated measured continuous outcomes (baseline, 4 months, 6 months). All statistical analyses on the secondary outcomes were conducted on an intention-to-treat basis, so that all participants were included according to original treatment assignment and analyzed regardless of adherence to treatment or dropout status. Incomplete data resulting from premature intervention dropouts was handled through the last observation carried forward method. In all secondary outcome analyses, important baseline covariates known to increase fall risk factors (age, sex, health status, history of falls, chronic conditions, level of physical activity) were incorporated into model testing.

To correct for multiple testing, the Bonferroni correction was made. Specifically, we had a total of three major sets of hypotheses that involved the outcomes of physical performance and cognitive function, which resulted in a total of 6 comparisons (TJQMBB vs. Stretching, Multimodal Exercise vs. Stretching) with 2 on each outcome, with an adjusted alpha value of 0.007 (0.05 / 7 comparisons) for each test to be considered statistically significant. All analyses were conducted using SPSS version 23 (IBM Corp, Armonk, NY) or Stata (StataCorp LP, release 13).

Power. Power calculations for the primary outcome—incidence of falls during 24 weeks of intervention between the two experimental exercise interventions (TJQMBB, Multimodal Exercise) relative to Stretching—were based on calculating the difference between two negative binomial rates using the method outlined by Zhu and Lakki.[19] The estimate for TJQMBB was derived from our prior trial[7] (which was comparable in study population, design, and intervention control) that showed a 55% reduction in the fall rate for Tai Ji Quan (within-person correlations ranged from Kendall's τ =0.2 to 0.4) compared to a stretching control. Estimates from the Lord et al. study[5] showed a reduction in fall rate of 22%-31% between Multimodal Exercise and a low-impact control program. On the basis of these empirical estimates, we conservatively estimated a reduction of at least 35% (effect size) across the 24-week period in the two intervention groups (TJQMBB, Multimodal Exercise) versus Stretching. This 35% reduction was derived as a compromise between the improvement in fall rates shown by the two studies

(55% vs. 31%) in which more robust older adults without specific fall risks were targeted for intervention. It also represented a clinically meaningful change. Although a difference was anticipated in favoring TJQMBB, power was not calculated between TJQMBB and Multimodal due to the lack of a priori effect size estimates.

Assuming (a) a mean fall rate of approximately 2 with a low-impact control condition,[5,7] (b) 0.80 power, (c) a 2-sided, 0.05 significance level, (d) administrative censoring (i.e., censoring occurs when the study ends or when participants are lost to follow-up), and (e) an estimate of the dispersion parameter of 1, a sample size of 567 participants (189 per group) was required to detect a 35% reduction in the falls incidence (an IRR of 0.65) between either of the two intervention groups relative to Stretching. With an estimated 15% attrition, 666 total participants were planned.

With a final sample of at least 222 in each intervention group, we calculated power of over 95% to detect a between-group mean difference (TJQMBB vs. Stretching, Multimodal Exercise vs. Stretching) of 2.0 inches in Functional Reach,[7,20] 2 seconds in I-TUG, and 1.0 point change in SPPB score.[16] Calculations were based on the adjusted alpha level of 0.007 and within person correlation of 0.8.

References Cited

1. Podsiadlo D, Richardson S. The Timed “Up & Go”: A test of basic functional mobility for frail elderly persons. *J Am Geriatr Soc* 1991;39:142-148.
2. Shumway-Cook, A, Brauer, S, et al. Predicting the probability for falls in community-dwelling older adults using the Timed Up & Go Test. *Phys Ther* 2000;80(9): 896-903.
3. Folstein MF, Folstein SE, McHugh PR. Mini-Mental State: A practical method for grading the cognitive state of patients for the clinician. *J Psychiatrics Res*, 1975, 12(3):189–98.
4. Li F. Transforming traditional Tai Ji Quan techniques into integrative movement therapy - Tai Ji Quan: Moving for Better Balance. *J Sport Health Sci*. 2014;3:9-15.
5. Lord SR, Castell S, Corcoran J, Dayhew J, Matters B, Shan A, Williams P. The effect of group exercise on physical functioning and falls in frail older people living in retirement villages: A randomized, controlled trial. *J Am Geriatr Soc* 2003 Dec;51(12):1685-1692.
6. Stevens JA, Burns E. *A CDC Compendium of Effective Fall Interventions: What Works for Community-Dwelling Older Adults* (2nd Edition). Available at: http://www.cdc.gov/HomeandRecreationalSafety/Falls/compendium.html. Accessed on February 1, 2018.
7. Li F, Harmer P, Fisher KJ, McAuley E, Chaumeton N, Eckstrom E, Wilson NL. Tai Chi and fall reductions in older adults: A randomized controlled trial. *J Gerontol: Med Sci* 2005;60:187-194.
8. Administration on Aging. Disease Prevention and Health Promotion Services (OAA Title IIID). Available at: https://www.aging.ca.gov/ProgramsProviders/AAA/Disease_Prevention_and_Health_Promotion/ Accessed on February 1, 2018.
9. Li F, Harmer P, Fitzgerald K, et al. Tai Chi and postural stability in patients with Parkinson’s Disease. *N Engl J Med* 2012;366, 511-519.
10. Craig CL, Marshall AL, Sjöström M, Bauman AE, Booth ML, Ainsworth BE, et al. International physical activity questionnaire: 12-country reliability and validity. *Med Sci Sports Exerc* 2003;35(88):1381-1395.
11. Tinetti ME, Inouye SK, Gill TM, Doucette JT. Shared risk factors for falls, incontinence, and functional dependence. Unifying the approach to geriatric syndromes. *JAMA* 1995;273:1348-53.
12. Robertson MC, Campbell AJ, Gardner MM, Devlin N. Preventing injuries in older people by preventing falls: A meta-analysis of individual-level data. *J Am Geriatr Soc* 2002;50:905-911.
13. Duncan PW, Weiner DK, Chandler J, et al. Functional reach: A new clinical measure of balance? *J Gerontol A Biol Sci Med Sci* 1990;45:192-197.
14. Salarian A, Horak F, Zampieri C, et al. iTUG, a sensitive and reliable measure of mobility. *IEEE Trans Neural Syst Rehabil Eng* June 2010; 18(3): 303–310.
15. Zampieri C, Salarian A, Carlson-Kuhta P, Nutt JG, Horak FB. Assessing mobility at home in people with early Parkinson’s disease using an instrumented Timed Up and Go test. *Parkinsonism and Related Disorders* 2011;17:277-280.
16. Guralnik JM, Simonsick EM, Ferrucci L, Glynn RJ, Berkman LF, Blazer DG, Scherr PA, Wallace RB. A short physical performance battery assessing lower extremity function: Association with self-reported disability and prediction of mortality and nursing home admission. *J Gerontol Med Sci* 1994; 49(2):M85-M94.
17. National Institute on Aging. Assessing physical performance in the older patient. Available at: http://www.grc.nia.nih.gov/branches/leps/sppb/. Accessed January 13, 2015.
18. Berstein IH, Lacritz L, Barlow CF, Weiner MF, DeFina LF. Psychometric evaluation of the Montreal Cognitive Assessment (MoCA) in three diverse samples. *Clinical Neuropsychologist* 2011;25:(1), 119-126.

19. Zhu H, Lakkis H. Sample size calculation for comparing two negative binomial rates. *Statistics in Med* 2014;3:367-387.
20. Li F, Harmer P, Stock R, Fitzgerald K, Stevens J, Gladieus M, Chou L-S, Carp K, Voit J. Implementing an evidence-based fall prevention program in an outpatient clinical setting. *J Am Geriatr Soc* 2013;61:2142-2149. DOI: 10.1111/jgs.12509.
